# Association of ABO blood group with severe *falciparum* malaria in adults: case control study and meta-analysis

**DOI:** 10.1186/1475-2875-11-S1-P75

**Published:** 2012-10-15

**Authors:** Aditya K Panda, Santosh K Panda, Aditya N Sahu, Rina Tripathy, Balachandran Ravindran, Bidyut K Das

**Affiliations:** 1 Infectious Disease Biology Group, Institute of Life Sciences, Bhubaneswar, Odisha, 751023, India; 2 Department of Medicine, S.C.B. Medical College, Cuttack, Odisha, 753007, lndia; 3 Department of Biochemistry, S.C.B. Medical College, Cuttack, Odisha, 753007, India

## Background

Erythrocyte-associated antigenic polymorphisms or their absence have perhaps evolved in the human population to protect against malarial infection. Studies in various populations consistently demonstrate that blood group ‘O’ confers resistance against severe falciparum infection. In India, Odisha state has one of the highest incidences of *Plasmodium falciparum* infection and contributes to the highest number of deaths by falciparum malaria. This study aims to evaluate the relationship between ABO blood group and severe malaria in an adult population at the tertiary care centre in Odisha.

## Methods

A total of 353 *P. falciparum* infected subjects and 174 healthy controls were screened for ABO blood group. Falciparum-infected individuals were categorized as severe malaria and uncomplicated malaria. Severe malaria was further clinically phenotyped into cerebral malaria, non-cerebral severe malaria and multi-organ dysfunction. A meta-analysis was performed to assess the role of ABO blood group in severe malaria.

## Results

Frequency of blood group ‘B’ was significantly higher in patients with severe malaria compared to the uncomplicated cases (P < 0.0001; OR = 4.09) and healthy controls (P < 0.0001; OR = 2.79). Irrespective of the level of clinical severity, blood group ‘B’ was significantly associated with cerebral malaria (P < 0.0001; OR = 5.95), multi-organ dysfunction (P < 0.0001; OR = 4.81) and non-cerebral severe malaria patients (P = 0.001; OR = 3.02) compared to the uncomplicated category. Prevalence of ‘O’ group in uncomplicated malaria (P < 0.0001; OR = 2.81) and healthy controls (P = 0.0003; OR = 2.16) was significantly high compared to severe malaria. Meta-analysis of previous studies, including the current one, highlighted the protective nature of blood group ‘O’ to severe malaria (P = 0.01). On the other hand, carriers of blood group ‘A’ (P = 0.04) and ‘AB’ (P = 0.04) were susceptible to malaria severity.

## Conclusions

Results of the current study indicate that blood group ‘O’ is associated with reduced and ‘B’ blood group with increased risk of development of severe malaria in Odisha, India. Meta-analysis also supports the protective nature of blood group ‘O’ from severe falciparum infection.

